# The Platform trial In COVID-19 vaccine priming and BOOsting (PICOBOO) booster vaccination substudy protocol

**DOI:** 10.1186/s13063-024-08456-4

**Published:** 2024-11-01

**Authors:** McLeod C, Dymock M, Flanagan KL, Plebanski M, Marshall H, Marsh J, Estcourt MJ, Ramsay J, Wadia U, Williams PCM, Tjiam MC, Blyth C, Subbarao K, Nicholson S, Faust S.N., Thornton RB, Mckenzie A, Snelling T, Richmond P

**Affiliations:** 1 Wesfarmers Centre of Vaccines and Infectious Diseases, Telethon Kids Institute, Nedlands, Australia; 2 Infectious Diseases Department, Perth Children’s Hospital, Nedlands, Australia; 3 School of Medicine, University of Western Australia, Crawley, Australia; 4 Tasmanian Vaccine Trial Centre, Clifford Craig Foundation, Launceston General Hospital, Launceston, TAS Australia; 5 School of Health Sciences, College of Health and Medicine, University of Tasmania, Launceston, TAS Australia; 6 School of Health and Biomedical Sciences, Royal Melbourne Institute of Technology University (RMIT), Melbourne, Australia; 7 Robinson Research Institute and Adelaide Medical School, The University of Adelaide, Adelaide, Australia; 8 Women’s and Children’s Health Network, North Adelaide, Australia; 9 Sydney School of Public Health, Faculty of Medicine and Health, University of Sydney, Sydney, Australia; 10 Centre for Child Health Research, The University of Western Australia, Crawley, Australia; 11 Department of Immunology and Infectious Diseases, Sydney Children’s Hospital Network, Sydney, Australia; 12 School of Women and Children’s Health, University of New South Wales, Sydney, Australia; 13 Division of Paediatrics, School of Medicine, University of Western Australia, Crawley, Australia; 14 Department of Microbiology, Pathwest Laboratory Medicine WA, QEII Medical Centre, Perth, Australia; 15 WHO Collaborating Centre for Reference and Research On Influenza, University of Melbourne, Parkville, VIC Australia; 16 Department of Microbiology and Immunology, University of Melbourne at the Peter Doherty Institute for Infection and Immunity, Melbourne, Australia; 17 Victorian Infectious Diseases Reference Laboratory, The Royal Melbourne Hospital at the Peter Doherty Institute for Infection and Immunity, Melbourne, Australia; 18 Department of Infectious Diseases, University of Melbourne at the Peter Doherty Institute for Infection and Immunity, Melbourne, Australia; 19 Southampton Clinical Research Facility and Biomedical Research Centre, National Institute of Health Research, University Hospital Southampton NHS Foundation Trust, Southampton, UK; 20 Faculty of Medicine and Institute for Life Sciences, University of Southampton, Southampton, UK; 21 General Paediatrics and Immunology Departments, Perth Children’s Hospital, Nedlands, Australia

**Keywords:** COVID-19, Booster vaccination, Vaccination, Immunisation, Adaptive platform trial, Policy, Pandemic

## Abstract

**Background:**

Coronavirus-2019 (COVID-19) vaccination in Australia commenced in February 2021. The first vaccines recommended for use were AZD1222 and BNT162b2, both delivered as a two-dose primary schedule. In the absence of sustained immunity following immunisation, recommendations for booster vaccination have followed. It is likely that periodic boosting will be necessary for at least some Australians, but it is unknown what the optimal booster vaccines and schedules are or for whom vaccination should be recommended.

**Methods:**

The *P*latform Trial *I*n *CO*VID-19 priming and *BOO*sting (PICOBOO) is a multi-site, multi-arm, randomised, Bayesian adaptive platform trial evaluating different booster vaccine interventions in immunocompetent children and adults, stratified by their primary vaccination schedule and age. Participants are randomised to receive one of three licensed COVID-19 booster vaccines available for use in Australia. PICOBOO aims to generate evidence about the immunogenicity, reactogenicity, and cross-protection of different booster vaccine strategies against severe acute respiratory syndrome coronavirus 2 (SARS-CoV-2) and its variants/subvariants. The protocol structure specifying PICOBOO is modular and hierarchical. We have previously published the PICOBOO core (master) protocol. Here, we detail the substudy protocol which outlines the study processes which are specific to PICOBOO participants enrolled in the booster vaccination substudy.

**Discussion:**

PICOBOO is an adaptive platform trial evaluating different COVID-19 booster vaccination strategies to generate evidence to inform immunisation practice and policy. The modular and flexible protocol structure is intended to enable investigators to respond with agility to new research questions as they arise, such as immunogenicity targeting emergent virus variants, and the immunogenicity and reactogenicity of new vaccines as they become available for use.

**Trial registration:**

Australian and New Zealand Clinical Trials Register ACTRN12622000238774; registered on 10/02/2022. Protocol V8.0_23112023.

## Introduction

### Background and rationale {6a}

Primary COVID-19 vaccination in Australia commenced in February 2021, and recommendations for booster vaccination have followed in select populations. Vaccines recommended for primary vaccination in Australia included AZD1222 and mRNA vaccines (BNT162b2 or mRNA-1273) as a two-dose primary schedule in immunocompetent individuals. The need for periodic boosting, including across age groups and in vulnerable sub-populations, is the subject of ongoing debate globally [1]. The durability of protection offered by vaccination is likely to be influenced by the host, including exposure to severe acute respiratory syndrome coronavirus 2 (SARS-CoV-2), the circulating strains of the virus [1, 2], the vaccine dose, dosing interval, and type of vaccine administered, and the differential impacts of homologous versus heterologous schedules on immune responses (antibodies, B cells, CD4 + and CD8 + T cells) [3, 4].

The *P*latform Trial *I*n *CO*VID-19 priming and *BOO*sting (PICOBOO) is a randomised, Bayesian adaptive platform trial that is generating evidence of the immunogenicity, reactogenicity, and cross-protection of different booster vaccination strategies against SARS-CoV-2 and its variants/subvariants in immunocompetent adolescents and adults. This trial will also evaluate how these immune responses correlate with protection from infection and disease and how this is impacted by natural infection. PICOBOO has been designed in consultation with the National Community Advisory Group for COVID-19 research and members of national COVID-19 policymaking bodies and is intended to inform practice and policy in Australia.

**Fig. 1:**
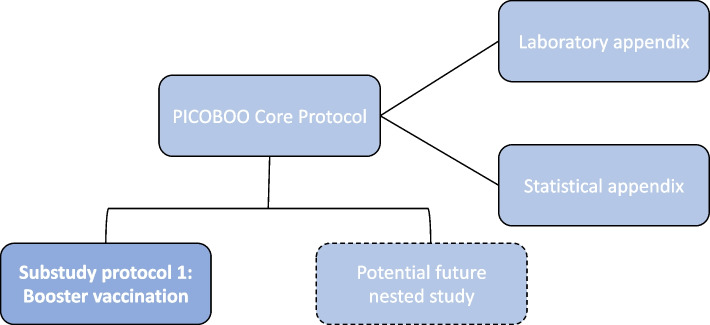
PICOBOO booster vaccination substudy protocol in relation to other study documents

The documentation specifying PICOBOO is modular and hierarchical (Fig. 1). The Core Protocol describes study procedures that apply to all participants and aspects of the trial and is presented elsewhere [5]. Here, we present the booster vaccination substudy protocol (SSP), which details the outcomes, endpoints, and study processes that relate specifically to participants enrolled in the booster vaccination substudy.

### Objectives {7}

The primary objective of the PICOBOO booster vaccination substudy is to generate high-quality evidence about the immunogenicity of different COVID-19 booster vaccination strategies against SARS-CoV-2 and its variants/subvariants in immunocompetent hosts, stratified by the primary vaccination history and age group.

### Trial design {8}

The PICOBOO booster vaccination substudy is a randomised, Bayesian adaptive platform trial. It will allow the introduction of new vaccines/schedules for evaluation in this platform and/or to remove vaccines in response to the emergence of external data or changes in immunisation policy in Australia, while preserving the integrity of the trial.

## Methods: participants, interventions, and outcomes

### Study setting {9}

This study is currently recruiting at three sites across Australia; these include Telethon Kids Institute (Western Australia), the Women’s and Children’s Hospital (South Australia), and Launceston General Hospital (Tasmania).

### Eligibility criteria {10}

To be eligible a person must:

1. Meet eligibility criteria for the PICOBOO platform trial as outlined in the PICOBOO core protocol [5]
2. Be ≥ 12 years old
3. Have undergone primary COVID-19 vaccination with:

(i) Two doses of AZD1222 and be 50 years or over OR
(ii) Two doses of BNT162b2 and be less than 70 years OR
(iii) Two doses of mRNA-1273 and be aged between 12 and 18 years old

A person is *not* eligible if they:

1. Have received a COVID-19 booster vaccine in the preceding 3 months*
2. Are contraindicated to receive any of the COVID-19 study vaccines, e.g. have a history of anaphylaxis to a vaccine component

*If the Australian Technical Advisory Group on Immunisation (ATAGI) advises an alternative window for COVID-19 boosting vaccinations for eligible participants, this criterion will be reviewed.

Participants already enrolled in the PICOBOO adaptive platform trial may be eligible for re-randomisation to receive additional COVID-19 booster vaccine doses, provided the eligibility criteria detailed in the PICOBOO core protocol [5] and the PICOBOO booster vaccination substudy protocol are met.

### Who will take informed consent? {26a}

Informed consent will be obtained by means of written or electronic signature which will be dated and countersigned by the research staff member who obtained it. Additional details regarding the consent process are detailed in in the PICOBOO Core Protocol [5].

### Additional consent provisions for collection and use of participant data and biological specimens {26b}

As detailed in the PICOBOO Core Protocol [5], participants and/or their legal guardians provided consent for blood and saliva samples to be tested and stored for COVID-19 research (including genetic studies) at participating laboratories. Participants have additionally provided consent for de-identified data and samples to be used for future research by participating institutions and third-party organisation(s).

## Interventions

### Explanation for the choice of comparators {6b}

All vaccines evaluated in this trial will be approved for use in the target age group by Australia’s Therapeutic Goods Administration (TGA) (including for emergency use) or an equivalent regulatory agency. However, vaccines need not be recommended by ATAGI for boosting for all age groups or specific populations under study. This trial is operating under a TGA clinical trial notification (CTN).

Vaccines investigated at trial commencement may be superseded by updated vaccine formulations as they become available for use in Australia (e.g. vaccines targeting new SARS-CoV-2 variants). To ensure the contemporary relevance of the trial data, investigational vaccines will be introduced or removed from the platform uniformly across all study sites at the discretion of the trial steering committee (TSC), with oversight provided by the data safety monitoring committee (DSMC).

### Intervention description {11a}

Participants within each stratum will be randomised to receive a single COVID-19 booster vaccine. Each stratum is defined by primary vaccination schedule (two doses of AZD1222 (Vaxzevria, AstraZeneca), BNT162b2 (Comirnaty, Pfizer), or mRNA-1273 (Spikevax, Moderna) and age cohort (12– < 18, 18– < 50, 50– < 70, or 70 + years of age).

At the time of publication, nine COVID-19 vaccines have been evaluated in the PICOBOO platform. BNT162b2, mRNA-1273, and NVX-CoV2372 (Nuvaxovid, Novavax) were evaluated at trial commencement. BNT162b2 and mRNA-1273 were removed for evaluation on January 4, 2023. Tozinameran/riltozinameran (Comirnaty original/Omicron BA.1, Pfizer) and elasomeran/imelasomeran (Spikevax bivalent Original/Omicron, Moderna) were introduced for evaluation on January 5, 2023, and removed for evaluation on June 4, 2023. Tozinameran/famtozinameran (Comirnaty Original/Omicron BA.4–5, Pfizer) and elasomeran/davesomeran (Spikevax bivalent Original/Omicron BA.4–5, Moderna) were introduced for evaluation on June 5, 2023, and removed for evaluation on January 8, 2024. Raxtozinameran (Comirnaty Omicron XBB.1.5, Pfizer) and andusomeran (SPIKEVAX XBB.1.5, Moderna) were introduced to the platform on January 9, 2024. Dosing and administration information is described in further detail, below:

1. BNT162b2

This mRNA vaccine encodes the full-length SARS-CoV-2 spike protein. The dose for individuals ≥12 years old is 30 micrograms (μg) in 0.3mL of the diluted vaccine delivered by intramuscular injection. Each multidose vial contains 6 doses.

2. mRNA-1273

A lipid nanoparticle encapsulated mRNA which encodes the full-length SARS-CoV-2 spike protein modified with 2 proline substitutions within the heptad repeat 1 domain. The booster dose is 50μg (0.25mL) for individuals aged >12 years old delivered as an intramuscular injection. Each multidose vial contains 20 doses.

3. NVX-CoV2373

A nanoparticle vaccine. It is constructed from the full-length wild-type pre-fusion trimers of SARS-CoV2 spike glycoprotein and is co-formulated with a saponin-based adjuvant, Matrix-M1™. The booster dose is 5μg (0.5mL) for individuals aged >12 years old delivered as an intramuscular injection. Each multidose vial contains 10 doses.

4. Tozinameran/riltozinameran

This mRNA vaccine comprises tozinameran and riltozinameran, the later encoding the viral spike protein of SARS-CoV-2 Omicron BA.1. The dose for individuals >12 years of age is 15μg tozinameran and 15μg riltozinameran in 0.3mL delivered as an intramuscular injection. Each multidose vial contains 6 doses.

5. Elasomeran/imelasomen

This vaccine contains mRNA coding for the original mRNA-1273 spike glycoprotein as well as the altered version mRNA-1273.529, based on the original mRNA-1273 vaccine but containing mRNA that encodes for the B.1.1.529-matched S glycoprotein. The dosage of mRNA-1273.214 is 25μg of elasomeran and 25μg of imelasomeran in 0.5mL for individuals >12 years old delivered as an intramuscular injection. Each multidose vial contains 5 doses.

6. Tozinameran/famtozinameran

This vaccine comprises mRNA including tozinameran and riltozinameran, the later encoding the viral spike (S) protein of SARS-CoV-2 Omicron BA.1. The dose of Comirnaty bivalent Original/Omicron BA.1 for boosting is 15 of tozinameran and 15 of riltozinameran contained in 0.3mL of the diluted vaccine for individuals aged ≥12 years old delivered as an intramuscular injection. Each multidose vial contains 6 doses.

7. Elasomeran/davesomeran

This vaccine comprises mRNA including elasomeran and of davesomeran, the later encoding the viral spike (S) protein of SARS-CoV-2 (Omicron BA.4-5). The S proteins of the SARS-CoV-2 Omicron variant lineages BA.4 and BA.5 are identical. The dosage of mRNA-1273.222 is 25μg of elasomeran and 25μg of davesomeran in 0.5mL for individuals >12 years old delivered as an intramuscular injection. This formulation will be available as either a multidose vial containing 5 doses, or as a single dose per vial.

8. Raxtozinameran

This is an mRNA vaccine which encodes the viral spike protein of SARS-CoV-2 Omicron XBB.1.5. One dose (0.3 mL) contains 30μg (≥12 years) or 10μg (5 to <12 years) of raxtozinameran delivered as an intramuscular injection. A single vial contains one dose. The formulation for individuals >12 years of age is available as a multi-dose vial, containing 6 doses. The formulation for use in children 5 to <12 years of age is available in a single dose vial.

9. Andusomeran

This is an mRNA vaccine which encodes the viral spike protein of SARS-CoV-2 Omicron XBB.1.5. One dose contains 50μg of andusomeran in 0.5mL for individuals >12 years old delivered as an intramuscular injection. A single vial contains one dose.

### Criteria for discontinuing or modifying allocated interventions {11b}

A maximum of 50 participants per intervention per intervention strategy (booster dose number) per stratum will be recruited. Randomisation to an intervention within an intervention strategy and stratum will be ceased prior to enrolment of 50 participants if required because of external factors (such as updates to ATAGI vaccine recommendations) or if a pre-specified statistical criterion is exceeded at a pre-specified interim analysis. The statistical criterion is based on the precision of the primary estimate for each intervention assessed in the intervention strategy and stratum as detailed in a separate Statistical Appendix [6].

### Strategies to improve adherence to interventions {11c}

Investigational vaccines are delivered as a single dose on the day of randomisation.

### Relevant concomitant care permitted or prohibited during the trial {11d}

As detailed in the core Protocol [5], individuals were permitted to receive their regular medications if they participated in the trial in addition to paracetamol if required, after vaccine administration.

### Provisions for post-trial care {30}

As detailed in the PICOBOO Core Protocol [5], participants will receive usual supportive care following vaccination, as per standard Australian immunisation practice. Specifically, participants will be observed for a minimum of 15 min, and supportive treatment for the management of acute hypersensitivity reactions (e.g. anaphylaxis) will be administered, if required.

### Outcomes {12}

Outcomes reported for all participants enrolled in the booster vaccination substudy will include the log_10_ concentration of SARS-CoV-2 anti-spike immunoglobulin (IgG) at the time points indicated in Table 1, clinical outcomes, and reactogenicity and safety outcomes, as detailed in the core protocol [5]. Additional pre-specified immunological tests will be performed on a dedicated subset, comprising the first 20 participants per booster dose number per stratum to provide samples within the window period at visit 3 (day 28) [6]. Outcomes and endpoints for participants enrolled in the booster vaccination substudy are detailed in Table 1. The availability of new assays and/or funding constraints may impact on immunological testing as the trial progresses.

**Table 1:** Outcomes and endpoints for participants in the booster vaccination substudy

Outcomes/endpoints*	
***Immunological***	
Concentration of anti-spike Immunoglobulin G (IgG antibody) against SARS-CoV-2	Day 7, 24, 84/180**, 365
Concentration of neutralising SARS-CoV-2 antibodies against SARS-CoV-2***	Day 24, 84/180**, 365
The percentage inhibition of SARS-CoV-2***	Day 28, 84/180**, 365
The number of IFN-γ^b^ spot forming cells per 10^6^/L peripheral blood mononuclear cells, following in vitro stimulation with SARS-CoV-2 spike overlapping pools of lyophilized peptides, consisting mainly of 15-mer sequences with 11 amino acids overlap***	Day 7, 84/180**, 365
Mucosal salivary IgA^c^ and IgG^d^	Day 28, 84/180**, 365
Presence of anti-nucleocapsid antibodies	Day 7, 28, 84/180**, 365
***Reactogenicity/safety***	
Participant or carer-reported local and systemic reactions assessed daily up to day 7 graded as no reaction, mild, moderate, severe, or life-threatening	Days 1–7
Any participant or carer-reported solicited and unsolicited AE^e^ up to ~ 28 days	Day 28
Hospitalisation resulting from AEFI^f^ up to ~ 28 days	Day 28
Any SAR^g^ thought to be causally related to the study intervention	Variable
***Clinical***	
Any PCR^h^-confirmed SARS-CoV-2 OR RAT^i^ positive result for SARS-CoV-2 up to day 720	Variable
Any PCR^h^-confirmed SARS-CoV-2 infection up to day 720	Variable
Any PCR^h^-confirmed wild-type or predominant circulating variant SARS-CoV-2 infection up to day 720	Variable
Any hospitalisation (days) for management of PCR^h^-confirmed or RAT^i^-positive SARS-CoV-2 infection up to day 720	Variable
Any participant or carer-reported days off work, study, or usual activities (days) due to PCR^h^-confirmed or RAT^i^-positive SARS-CoV-2 infection up to day 720	Variable

^*^Relative to randomisation

^**^For adolescent participants and adult participants receiving a 3rd or subsequent (5th dose) booster, visit 4 (day 84) will be substituted for day 180

^***^This will be performed separately for ancestral SARS-CoV-2 and the predominant circulating variant

^a^Primary endpoint

^b^IFN- γ: interferon-γ

^c^IgA: Immunoglobulin A

^d^IgG: Immunoglobulin G

^e^AE: Adverse event

^f^AEFI: Adverse event following immunisation

^g^SAR: Serious adverse reaction

^h^PCR: Polymerase chain reaction

^i^RAT: Rapid antigen test

### Participant timeline {13}

The schedule of events detailing enrolment, the intervention, and study visits are detailed in Table 2.

**Table 2:**
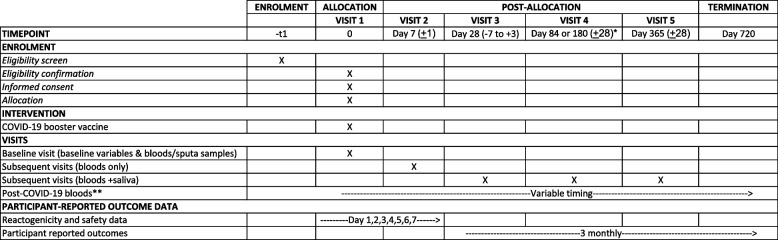
Timeline for enrolment, allocation and post-allocation events and trial termination

*For adolescent participants and participants receiving a 5th or subsequent COVID-19 booster vaccination, visit 4 will be conducted on Day 180

**To be collected a minimum of 7 days after confirmed infection, or as soon as quarantine restrictions abate

For re-randomised participants, study visits 1–5 will be repeated. Any visits remaining from the previous randomisation will not be performed. Collection of participant-reported outcome data will recommence from the point of re-randomisation.

### Sample size {14}

The maximum planned recruitment per intervention per stratum for each booster dose is 50 participants. The pre-planned adaptations include ceasing recruitment to a booster dose within a stratum when the precision threshold is met for the primary estimand across all interventions and are detailed in the Statistical Appendix [6].

### Recruitment {15}

As detailed in the PICOBOO Core Protocol [5], potential screening avenues to identify eligible participants include during vaccination/healthcare visits and advertising material via community locations, research and consumer networks, and social media and via targeted recruitment via Services Australia Medicare mailouts or short-message services delivered via healthcare providers (e.g. via the SMARTVAX network).

## Assignment of interventions: allocation

### Sequence generation {16a}

A sequence of intervention assignments will be generated by an un-blinded trial statistician using random permuted blocks for each booster dose within each stratum using computer software with a validated random number generator and equal allocation for all booster interventions.

### Concealment mechanism {16b}

As detailed in the PICOBOO Core Protocol [5], an unblinded research nurse will obtain the next contiguous stratum allocation (i.e. the lowest available randomisation number) from the study REDCap database on the day of randomisation.

### Implementation {16c}

As detailed in the PICOBOO Core Protocol [5], at vaccination, two unblinded members of the research team will check and dispense the study vaccine for administration. Pre-filled syringes containing one of the COVID-19 booster vaccines approved for use will be covered with opaque tape and concealed until ready for administration. Prior to opening the box, the participant will be asked to look away. The vaccine intervention will be administered in accordance with routine immunisation practices stipulated per Australian guidelines.

## Assignment of interventions: blinding

### Who will be blinded {17a}

Participants will be blinded to the specific COVID-19 vaccination received, at least until after the primary estimand data are collected. Further details are provided in the PICOBOO Core Protocol [5], At this time, details regarding vaccination will be uploaded to the Australian Immunisation Register, where it will be possible for participants to access their individual vaccination history, if desired.

### Procedure for unblinding if needed {17b}

The corresponding site principal investigator will provide authorisation for unblinding if compelling reasons arise. Further details are provided in the PICOBOO Core Protocol [5].

## Data collection and management

### Plans for assessment and collection of outcomes {18a}

As detailed in the PICOBOO Core Protocol [5], data will be collected on hard or electronic case report forms (eCRF), including (i) demographic data, (ii) COVID-19 vaccination history, (iii) previous medical history, (iv) anthropometric data (including height and weight), (v) laboratory data, and (vi) participant/carer-reported outcomes.

### Plans to promote participant retention and complete follow-up {18b}

Participants will be contacted and reminded to attend for their follow-up visits. We will use all available data on any patients who are lost to follow-up. If a participant withdraws, we will use all data collected up until the time of withdrawal unless they explicitly request their data to be removed.

### Data management {19}

Data will be collected into a secure REDCap database, hosted by the sponsor. Further details regarding data management and security are provided in the PICOBOO Core Protocol [5],

### Confidentiality {27}

Robust measures will be taken to ensure confidentially for participants enrolled in the trial, as detailed in the PICOBOO Core Protocol [5].

### Plans for collection, laboratory evaluation, and storage of biological specimens for genetic or molecular analysis in this trial/future use {33}

As detailed in Table 2, blood and saliva samples will be collected at pre-specified time points. Blood will be separated into the sera, plasma, and peripheral blood mononuclear cells (PBMCs) at local sites prior to transportation to reference laboratories for processing. Granulocytes collected from baseline blood samples during gradient centrifugation will be used for deoxyribonucleic acid (DNA) extraction for human leucocytic antigen (HLA) I and II typing. This will be performed to evaluate susceptibility to and protection from SARS-CoV-2 infection and disease. Genomic DNA will be extracted using commercial kits, harmonised across sites. DNA will be aliquoted and stored as per standard operating procedures (SOPs).

## Statistical methods

### Statistical methods for primary and secondary outcomes {20a}

A Bayesian three-level hierarchical linear model will be used for the primary analysis as it is anticipated that immune responses may be mutually informative across COVID-19 vaccination dose, age groups, and potentially across messenger ribonucleic acid (mRNA) vaccine interventions [6]. The model estimates the posterior distribution of the mean log10 anti-spike SARS-CoV-2 IgG antibody against Ancestral SARSCoV-2 measured ~ 28 days after receipt of the assigned booster COVID-19 vaccine for each intervention and vaccination strategy in each stratum, denoted by vaccine history group and age group. Further detail can be found in the Statistical Appendix [6].

### Interim analyses {21b}

Interim analyses were pre-specified. The first analysis was performed after participants had completed 300 vaccination events and 21–31 days follow-up post-randomisation. Further details regarding interim analyses detailed in the Statistical Appendix [6].

### Methods for additional analyses (e.g. subgroup analyses) {20b}

Any analyses not specified in the Statistical Appendix [6] will be designated as exploratory.

### Methods in analysis to handle protocol non-adherence and any statistical methods to handle missing data {20c}

Immune responses and reactogenicity to COVID-19 vaccines will be assessed using a treatment policy strategy. Further detail regarding the analytical approach to handling intercurrent events is detailed in the Statistical Appendix [6].

### Plans to give access to the full protocol, participant-level data, and statistical code {31c}

Current versions of the PICOBOO core protocol [5], the Statistical Appendix [6], the PICOBOO substudy protocol, and the Laboratory Appendix will be accessible on the trial website (https://picoboo.com.au/). Decisions regarding the sharing of de-identified data and/or statistical code will be assessed by the PICOBOO TSC and will be conditional upon any necessary institutional and ethics approvals.

## Oversight and monitoring

### Composition of the coordinating centre and trial steering committee {5d}

The PICOBOO administrative structure detailed in the PICOBOO core protocol applies to all nested platform substudies [5].

### Composition of the data monitoring committee, its role and reporting structure {21a}

As detailed in the PICOBOO Core Protocol [5], a data and safety monitoring committee (DSMC) will be appointed to provide safety oversight. The DSMC will have an advisory role as outlined in the DSMC Charter.

### Adverse event reporting and harms {22}

All serious adverse events (SAEs), adverse events of special interest (AESI), medically attended AEs (MAAEs), and adverse events (AEs) resulting in withdrawal occurring from day 0 to day 28 after randomisation will be recorded. From day 29 to day 720 after randomisation, all SAEs, AESIs, MAAEs, and AEs resulting in withdrawal that are found to be related to the study vaccine or study procedures will be reported. The processes for assessing and reporting safety are detailed in the PICOBOO Core Protocol [5].

### Frequency and plans for auditing trial conduct {23}

Monitoring will be conducted according to a risk-stratified approach as detailed in the PICOBOO Core Protocol [5].

### Plans for communicating important protocol amendments to relevant parties (e.g. trial participants, ethical committees) {25}

Any substantial amendments to the PICOBOO protocol will require prior approval by the relevant ethics and governance regulatory bodies [5].

### Dissemination plans {31a}

The National COVID-19 CRG will provide guidance on the best methods for dissemination of information to participants and the broader community. The TSC will, as far as possible, make the protocol(s), laboratory appendix, statistical analysis plans, and non-identifying patient-level data available, to allow independent scientific scrutiny and validation of any published results.

## Discussion

We present the protocol for a randomised, Bayesian adaptive trial nested within the PICOBOO study platform; the trial aims to generate high quality evidence regarding the immunogenicity, reactogenicity, and cross protection offered by different COVID-19 booster vaccination strategies against SARS-CoV-2 and its variants/subvariants. The PICOBOO booster vaccination substudy is unique in terms of evaluating the impacts of multiple alternative COVID-19 vaccination strategies concurrently and sequentially, across different age groups, including in children. It is expected to generate evidence to shape immunisation practice and policy while also contributing to the growing body of evidence informing vaccination policy globally.

COVID-19 vaccines have led to substantial reductions in severe disease, hospitalisation, and death, with an estimated 19.8 million deaths averted in 2021 alone [7]. While periodic booster vaccination is likely to remain a core strategy for achieving protection against disease in at least some risk groups, there are limited data to inform the best ongoing strategies for vaccination. Specifically, it is unknown whether some vaccines are better than others and, if so, which homologous and/or heterologous vaccine strategies are best and in whom and what the optimal intervals between booster doses are. We aim to provide a detailed profile of the immunological and safety outcomes of different vaccines available for use in Australia to add to the available published literature to aid decision-making.

We expect that the trial’s Bayesian adaptive design will have three main benefits over conventional trial methods [8]. First, the flexibility to introduce new vaccines or schedules for evaluation as they become available, or to remove vaccines in response to changes to vaccine policy in Australia or the emergence of external data, while preserving the overall integrity of the trial. Second, the decision rules are designed to avoid over-recruitment to a stratum based on pre-specified precision criteria, facilitating timely decision-making. This is possible owing to improved statistical efficiency resulting from data sharing that is expected with the use of hierarchical Bayesian statistical models. Third, the trial structure will facilitate nesting of additional studies in the future, which is likely to be more efficient and cost-effective than conducting multiple trials independently.

While serum anti-spike immunoglobin G concentrations and anti-SARS-CoV-2 neutralising antibody titres have emerged as correlates of vaccine protection against symptomatic COVID-19 disease [9, 10], more data are required to delineate specific threshold titres that correlate with protection [11], including against variants of concern. Furthermore, additional work is required to elucidate correlates of protection based on other immunologic functions, such as effector memory and T cell function, which may play a role in controlling infection once established [9]. It is intended that the data generated from this trial will be combined with other data available internationally to further inform correlates of protection against COVID-19 infection and disease.

One issue that has garnered attention throughout the pandemic has been the timely dissemination of data and how this is impacted by the need for rigorous peer-review, ideally prior to the release of results [12]. The Bayesian statistical model used in this trial will be periodically updated as data accrue, even after recruitment to a particular booster intervention (e.g. third dose, fourth dose boosters) ceases within a stratum. Statistical reports detailing pre-specified analyses will be made available within the public domain on the trial website as soon as practicable following review by the trial statistical analysis team, the trial steering committee, and the DSMC. Results will also be disseminated periodically via peer-reviewed publications. To facilitate knowledge transfer, modification, and re-purposing, all trial processes, governance arrangements, and digital infrastructure have been developed in partnership with relevant stakeholders and based on FAIR data principles (findable, accessible, interoperable, reusable) [13].

The need for robust evidence to inform practice and policy in the face of a rapidly evolving pandemic has outstripped efforts to generate it quickly; consequently, decision-making has largely occurred ahead of the science. Moving forward, important vaccine policy questions must be addressed with agility to ensure that the recommended vaccination schedules will enhance population-level immunity while protecting vulnerable sub-populations. The PICOBOO booster vaccination substudy will fill critical knowledge gaps to optimise immunisation policy in Australia and elsewhere. Initial results were generated in the first quarter of 2023.

### Trial status

Current PICOBOO Core Protocol Version 15.0_28112023. Substudy Protocol: Booster vaccination V8.0_28112023. Recruitment commencement date: 29 March 2022. Recruitment is expected to be completed by Februrary 2026.

## Data Availability

Access to data will be granted to study Investigators and authorised representatives from the sponsor and the regulatory authorities to allow trial-related monitoring, audits, and inspections to occur. PICOBOO will also comply with relevant jurisdictional and academic requirements relating to access to data, as apply at the time that the data are generated.

## Abbreviations

ATAGI: Australian Technical Advisory Group on Immunisation
COVID-19: Coronavirus 2019
DSMC: Data safety monitoring committee
mRNA: Messenger ribonucleic acid
PICOBOO: *P*Latform trial *I*n *CO*VID-19 *BOO*sting
SARS-CoV-2: Severe acute respiratory syndrome coronavirus-2
TKI: Telethon Kids Institute
TGA: Therapeutic Goods Administration
TSC: Trial steering committee
